# Energy utilization in cattle with steady state and non-steady state methods: the importance of thermal neutrality

**DOI:** 10.1016/j.heliyon.2018.e00843

**Published:** 2018-10-05

**Authors:** A.L. Schaefer, K. Ominski, S. Thompson, G. Crow, C. Bench, J. Colyn, A. Rodas-Gonzalez, D. Maharjan, R. Bollum, N.J. Cook, J. Basarab, H. von Gaza

**Affiliations:** a Dept. AFNS, University of Alberta, Edmonton, Canada; b Dept. Animal Science, University of Manitoba, Winnipeg, Canada; c Agriculture and Agri-Food Canada, Lacombe, AB, Canada; d Alberta Agriculture, Lacombe, AB, Canada; e RandR Acres Sire Breeder, Airdrie, AB, Canada; f HVG Software Solutions, Edmonton, Canada

**Keywords:** Agriculture

## Abstract

The efficiency by which animals utilize dietary energy is fundamental to the cost of production for protein of animal origin and to the carbon footprint an animal industry has. Hence, the development of cost effective methodology for determining these measurements of efficiency is important. The objective of the present study was to investigate the use of infrared thermography in a rapid, non-steady state method for measuring energy loss in cattle. Data from 241 yearling bulls and steers as well as heifers and mature cows are presented. Infrared images were collected following a 24h feed withdrawal period. The infrared thermal response in these animals was significantly ranked (P < 0.03) with conventional measurements of feed efficiency using residual feed intake values for animals demonstrated to be within a thermal neutral zone. When animals were not within a thermal neutral zone there was no significant ranking. The data suggests that the use of a non-steady state approach using infrared thermography for identifying metabolic efficiency in animals may be a more rapid and less expensive method for identifying differences in energy utilization. The data also demonstrates the importance of maintaining thermal neutrality when measuring metabolic efficiency irrespective of the methodology.

## 1 Introduction

The efficiency by which we capture and utilize energy is fundamentally important to the carbon footprint for our societies and has a direct impact on global warming. This is true for industrialized sectors such as transportation and utility industries and it is also true for animal agriculture. As expressed by Shepherd (2014) and Moran and Wall (2014) global animal agriculture has a significant impact on carbon cycles, carbon dioxide emissions and overall anthropogenic green house gas emissions.

In addition to the carbon footprint, the efficiency by which domestic animals utilize energy for food production is fundamentally important for the affordability and sustainability of animal agriculture. This efficiency affects the cost of raising animals and therefore ultimately the cost of high quality food for consumers. As expressed by Capper (2014) as global human populations increase more animal protein needs to be produced using fewer resources and with a smaller carbon footprint. As suggested in the same review, Hermansen and Kristensen (2014) finding ways to improve feed conversions of animals is one way to reduce the carbon footprint for animal agriculture. In fact, managing feed conversion could well be considered an important social license requirement for the animal industries.

Finding ways to measure and manage energy utilization in animals has a long and interesting history. Measuring the requirements for retaining energy in animals was described as early as 1861 by Lawes and Gilbert (1861). These concepts were later refined using such measurements as the Kleiber ratio (Kleiber, 1975) which basically defined the growth rate divided by the mass or weight of an animal to the three quarter power. Another approach was to measure the actual oxygen consumption of an animal, or conversely CO_2_ production, referred to generally as indirect calorimetry (Young et al., 1975; Kleiber, 1975). While accurate, and in fact a gold standard for monitoring energy utilization in an animal, this method required the establishment of a steady physiological and metabolic state in an animal. Establishing such a state was time consuming and with limited availability of the specialized equipment this approach was often useful in a research setting only. One notable exception in this regard is the recently developed C-Lock technology (C-Lock Inc® Rapid City SD) (Arthur et al., 2017) which is capable of assessing cattle CO_2_ production in days as opposed to weeks and months with earlier equipment.

More recent attempts to monitor energy utilization in animals have employed a method referred to as a “residual feed intake” (Koch et al., 1963). The basic principle uses the actual measurement of feed intake of an animal measured with feed intake monitoring systems such as Growsafe ® (Basarab et al., 2003a, b) or Calan Broadbent gates ® (Farris et al., 2006). This feed intake measurement along with knowledge of the animal's growth is used to calculate the actual feed/growth values compared to the predicted feed/growth values as determined for example directly within the study group or by some national standard such as the Nutrient Requirements of Beef Cattle (NRC, 1996; NASEM, 2016). This residual Feed Intake or RFI method has been used to monitor animals with different genetic capabilities for growth efficiency. These methods and approaches have been reviewed by Archer et al. (2002), Johnson et al. (2003), Herd and Arthur (2009) and Nkrumah et al. (2004, 2006).

However, these methods require an obligatory steady state condition in the monitored animals for factors including disease states, environmental stability and stress free conditions. Furthermore, these methods require these conditions to exist for some 70–110 days in the case of cattle. With controlled laboratory conditions these steady state conditions can be obtained. By contrast, under field conditions, where in fact these methods are most often employed, obtaining such steady state conditions can be a challenge. Diseases, such as bovine respiratory disease can occur frequently and be undetected with the industry standard practice of “pen checking” (Schaefer et al., 2007, 2012). Moreover, the weather environment in which animals are studied can and usually does vary and can place the animal outside its thermal neutral zone (Webster et al., 1970; Young et al., 1989; Hahn, 1999; Thompson, 2015). If such conditions occur during a RFI test then these days are effectively lost from the test. If undetected or ignored and left in the data set such occurrences can only bias the resulting data.

In the interests of finding a practical solution to these aforementioned issues the current study was initiated to test the effectiveness of a non-steady state method to monitor energy efficiency in a domestic animal model. Non-steady state methods are commonly used in biological (medical and veterinary) science for monitoring metabolic and health conditions such as; a diabetic state using a glucose tolerance method (De Fronzo and Abdul-Ghani, 2011); to monitor pituitary and adrenal function using an ACTH challenge test or a dexamethasone suppression test (Chernecky and Berger, 2013) and used to measure kidney function (Finco, 1980) just to mention a few. The use of a non-steady-state approach to monitor growth and growth efficiency, however, is to date novel.

Infrared thermography or IRT is a direct measurement of an animal's energy loss. As such infrared thermography has been demonstrated to be significantly correlated with conventional measurements of energy utilization including the measurement of RFI under steady state conditions (Schaefer et al., 2005; Colyn, 2013; Montanholi et al., 2008, 2010).

We hypothesised that IRT could also be used to monitor the energy loss in an animal under non-steady state conditions. We hypothesised in the current study that inducing an animal to conserve energy, by withdrawal of feed for example, would cause an animal to display its genetic ability to do so. We hypothesised that a simple overnight (18h-24h) feed withdrawal would cause an animal to conserve energy if it was genetically able to do so. Alternatively, if the animal was less biologically able to conserve energy then it would not reduce its energy utilization to the same extent.

The objective of the current study therefore was to test the ability of IRT in a non-steady state model to measure the energy utilization in a group of animals with known differences in energy efficiency. In addition, a further objective of the current study was to examine the impact of thermal neutrality on the outcome of RFI measurements compared to a direct measure of energy exchange in an animal collected with infrared thermography. Thermal Neutrality or the thermal neutral zone (TNZ) is basically defined as the range of temperatures or zone whereby an animal does not need to expend energy either for heat production (lower critical temperature) or for cooling (upper critical temperature). This is sometimes referred to as the thermal comfort zone. Thermal neutrality is a relative measurement and can vary in an animal depending on a number of conditions including the age, body condition, type of weather factors such as humidity and weather history (Young et al., 1989). However, the TNZ is generally defined as being between 15–20 C wide (Hahn, 1999).

## 2 Materials and methods

### 2.1 Animals and management

Data are reported on 241 head of yearling bulls and steers as well a heifers and mature cows contained in seven study groups. These animals were studied either at the University of Manitoba Glenlea farm, the University of Alberta Dairy Research and Technology Centre, the Lacombe Research Centre (Agriculture and Agri-Food Canada), or at an Alberta Bull Test Centre (Cattleland, Strathmore, Alberta).

The animals at the University of Manitoba consisted of 59 yearling Angus bulls studied at two separate time periods (Jan–March (Thermal Neutral Zone conditions or TNZ) and again March–June (Non Thermal Neutral Zone Conditions or NTNZ)) and a separate group of 44 yearling Angus crossbred steers (NTNZ). All animals were treated humanely with studies approved under the animal care committee (University of Manitoba). The RFI studies were conducted to the same standards and operating procedures whereby animals received a cereal silage based diet which met or exceeded their growth and maintenance requirements as established by the National Academy of Science (2016). All cattle were provided with *ad libitum* access to clean water and environmental protection with wind fences and bedding. A standardized minimum 70 day feed intake study was utilized for all animals using Growsafe ® feed monitoring equipment (Basarab et al., 2007). The feed intake study was conducted prior to the measurement of the infrared facial scans. For weather monitoring, the use of an on site weather station data was used at the University of Manitoba. Environmental conditions recorded for the animals studied at the University of Manitoba trials were: January–March mean temperature of -14.9 ± 7.3 (SD), Range -25.2 to -4.5. Delta T of 20.7 and a mean relative humidity of 74% (TNZ). March–June mean temperature of 1.0 ± 10.1. Range 5.6 to -28.8. Delta T of 34.4 C and a mean relative humidity of 70% (NTNZ). Steers studied Feb–April mean temperature of -0.4 ± 8.2. Range -16.2 to 19.6 Delta T 35.8 C and a relative humidity of 66% (NTNZ). Following a 24h off feed period the cattle were brought into a handling facility and non invasive scans were collected in triplicate from a distance of 2 meters using a FLIR S60 or T450sc camera (FLIR. Boston MA).

The animals studied at the University of Alberta consisted of five mature dairy cows (Holstein) averaging 4.4 years of age. This was a preliminary opportunity to examine dairy animals with known differences in RFI values. These animals were dry, pregnant cows between 207 and 238 days of gestation. The animals were housed in an indoor tie stall facility and fed a total mixed ration formulated for dairy cattle (NRC, 2001) consisting of alfalfa-barley silage, rolled barley and corn. The cattle had *ad libitum* access to clean water. The facility temperature was 11.8 ± 2.7 C and relative humidity of 44 ± 9% (TNZ). These cows had been monitored for feed consumption and growth prior to the infrared studies by direct measurement of daily feed consumption with a weight scale. Both facial and coronary band images were collected non invasively in triplicate on the cattle in their tie stalls using a FLIR T450sc camera from a distance of 2 meters (FLIR, Boston MA). All animals were cared for according to the Canadian Council on Animal Care, 1993, Canadian Council on Animal Care, 2009 and the use of these animals in the off feed study had been approved by the University animal Care Committee.

Animals at the Lacombe Research Centre consisted of two study groups. One group consisted of 20 Angus X Hereford crossbred cows between 6-8 years of age. The cattle were all at approximately eight months gestation. The animals were fed a balanced alfalfa hay cube diet including a mineral/vitamin supplement which met or exceeded the recommended requirements for beef cattle (NRC, 1996). The cattle were housed in open pens measuring approximately 40 × 40 meters with a wind fence and bedding. There was a roof cover on one third of the pen. The cattle had clean straw bedding and *ad libitum* access to clean water. The residual feed intake for these animals had been determined over the previous 79 days using a Grow Safe ® feed monitoring system and standard operating procedures as described by Basarab et al. (2003a,b, 2007). Weather during the RFI tests was an average temperature of -5.9 ± 4.5C. Range 3.5 to -16.5. Delta T of 20C. The mean relative humidity was 75% (TNZ). On the days of infrared image collections the cattle were brought into a beef handling facility following a 24h off feed time period. The animals were placed into a smaller pen measuring approximately 4 × 4 meters and non invasive triplicate facial scans were collected on each animal at a distance of approximately 2 meters using a FLIR S60 camera (FLIR, Boston MA). All procedures followed the Canadian Council on Animal Care guidelines (2009) and were reviewed and approved by the Lacombe Research Centre Animal Care Committee. The second study group at the Lacombe Research Centre (one year following the first group) consisted of fourty two Angus X Herford or Angus heifers and cows between three and seven years of age. These animals were cared for and managed and data collected in a similar manner to the first group. This group of cattle was studied between December and February with a mean temperature of −7.6 ± 5.6 C. Delta T of 28.9 C. The mean relative humidity was 80 % (NTNZ).

Animals at the Bull Test Facility (Cattle Land Strathmore AB. NTNZ) were provided by a commercial cattle breeder (R and R Acres, Airdrie. AB). The animals studied consisted of 26 yearling crossbred Limousin, Simmental and Angus bulls raised by R and R Acres. All animals were raised under the Code of Practice for the Care and Handling of Beef Cattle (2013 National Farm Animal Care Council, Canadian Cattlemen's Association, Calgary, AB) and the Strathmore test facility is a Canadian Counsel on Animal Care approved facility. For the growth efficiency study the cattle received a barley sileage ration which met or exceeded the nutrient requirements for beef cattle (NRC, 1996). The measurement of feed intake was again accomplished with the use of a Grow Safe ® feed monitoring system (Grow Safe, Airdrie AB). The cattle were housed in open pens measuring approximately 30 × 30 meters with wind fence protection and straw bedding. The animals also had access to *ad libitum* water. For the Strathmore Alberta site the nearest Environment Canada weather station was located at the Calgary Airport. The mean temperature for the RFI test period was 12.3 ± 4.8 C. The Range of temperature was 3.2–23 with a delta T of 19.8 C. The mean relative humidity was 59% (TNZ).

The standard operating procedures for all research facilities required the vaccination of all cattle for IBR, PI3, BVD, haemophilus, pasteurella and clostridial organisms. In addition, treatment of the animals for internal and ecto-parasites was also standard practice.

**Fig. 1:**
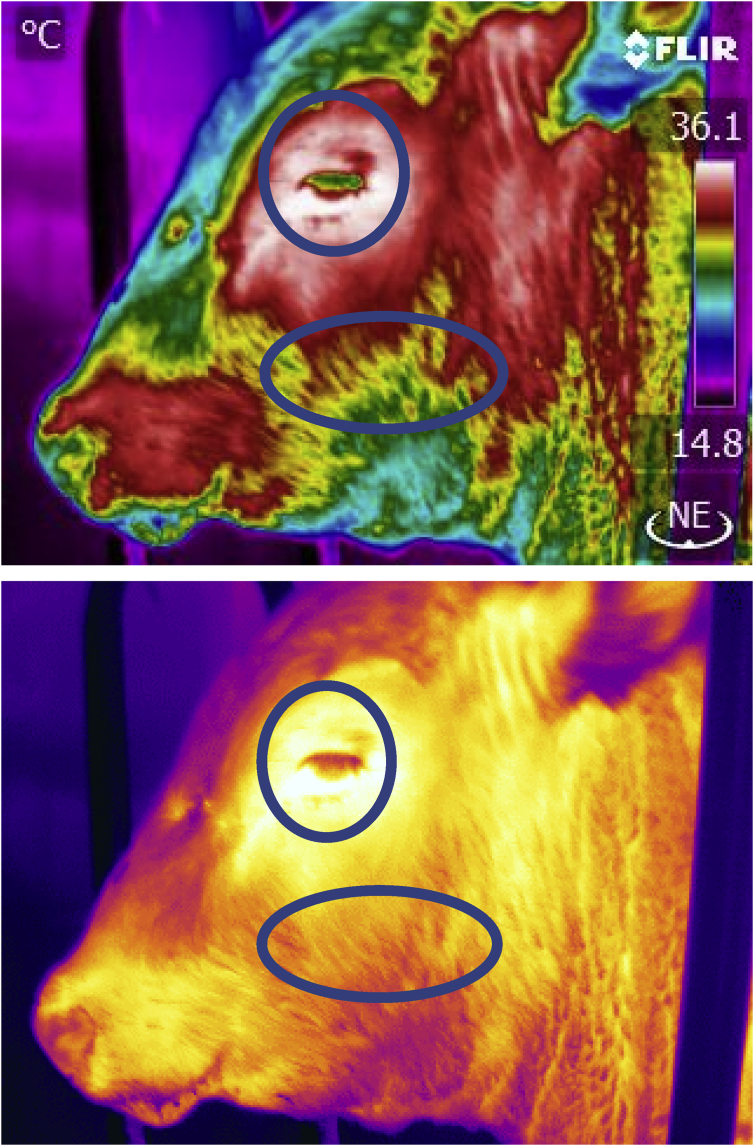
Infrared Thermograph of steer with a high orbital (eye) temperature of 37.5 C and high cheek mean temperature of 31.5 C. Areas used for orbital and cheek thermal values are depicted in rainbow palate (top) or lava palate (bottom).

**Fig. 2:**
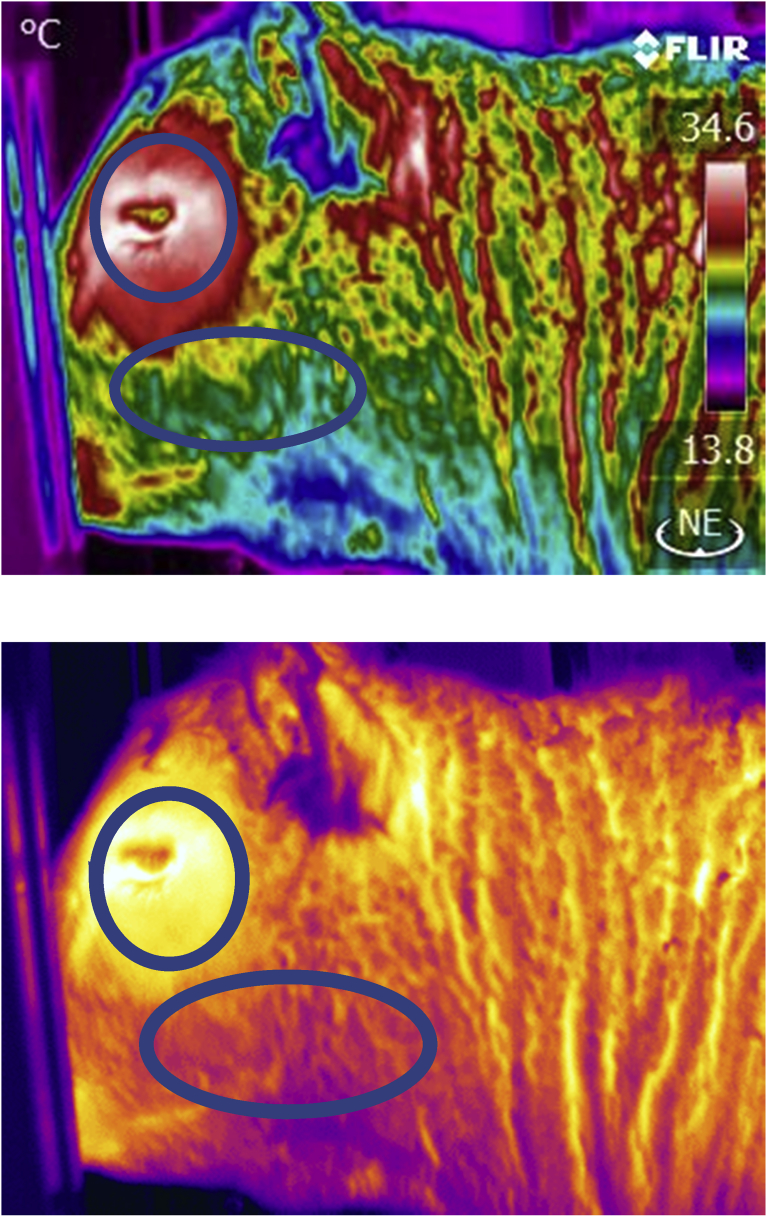
Infrared Thermograph of a steer with a normal eye temperature of 35.9 and normal cheek mean temperature of 28.8 C. Areas scanned are depicted in rainbow palate (top) or lava palate (bottom).

All cattle were scanned with infrared thermography cameras either before or following their RFI test. Basic Standard Operating Procedures for the infrared scanning have been described by Schaefer et al. (2018). The infrared hand held cameras used were either a model S60 or T450sc (FLIR Comp. Boston. MA). Both cameras are high resolution (320 × 240 pixel) instruments with a sensitivity of ±0.1 C and were calibrated according to specifications before and during the data collections. Values for atmospheric temperature and relative humidity used in the calibration were collected with a Kestrel model 3000 digital weather meter (Boothwyn PA. USA). Orbital or eye as well as cheek images were collected at a distance of 2 m at 90° to the animals (Figs. 1 and 2). An emissivity value of 0.98 was used for all animals. To compare all animal thermal values the infrared values were corrected or adjusted to the same scale by using a regression formula Y = b + ax where y = adjusted temperature, b = 23.3, a = 0.365, x = observed infrared temperature (r squared = 0.81). This regression formula was based on normal values from 570 cattle temperature observations collected over the temperature range used in the study.

All animals at the growth assessment facilities were weighed two consecutive days at the start of the study, again at mid test and also at the end of the test feeding period. The individual animal feed intake during the study was monitored in the Grow Safe ® bunks as described by Wang et al. (2006).

**Fig. 3:**
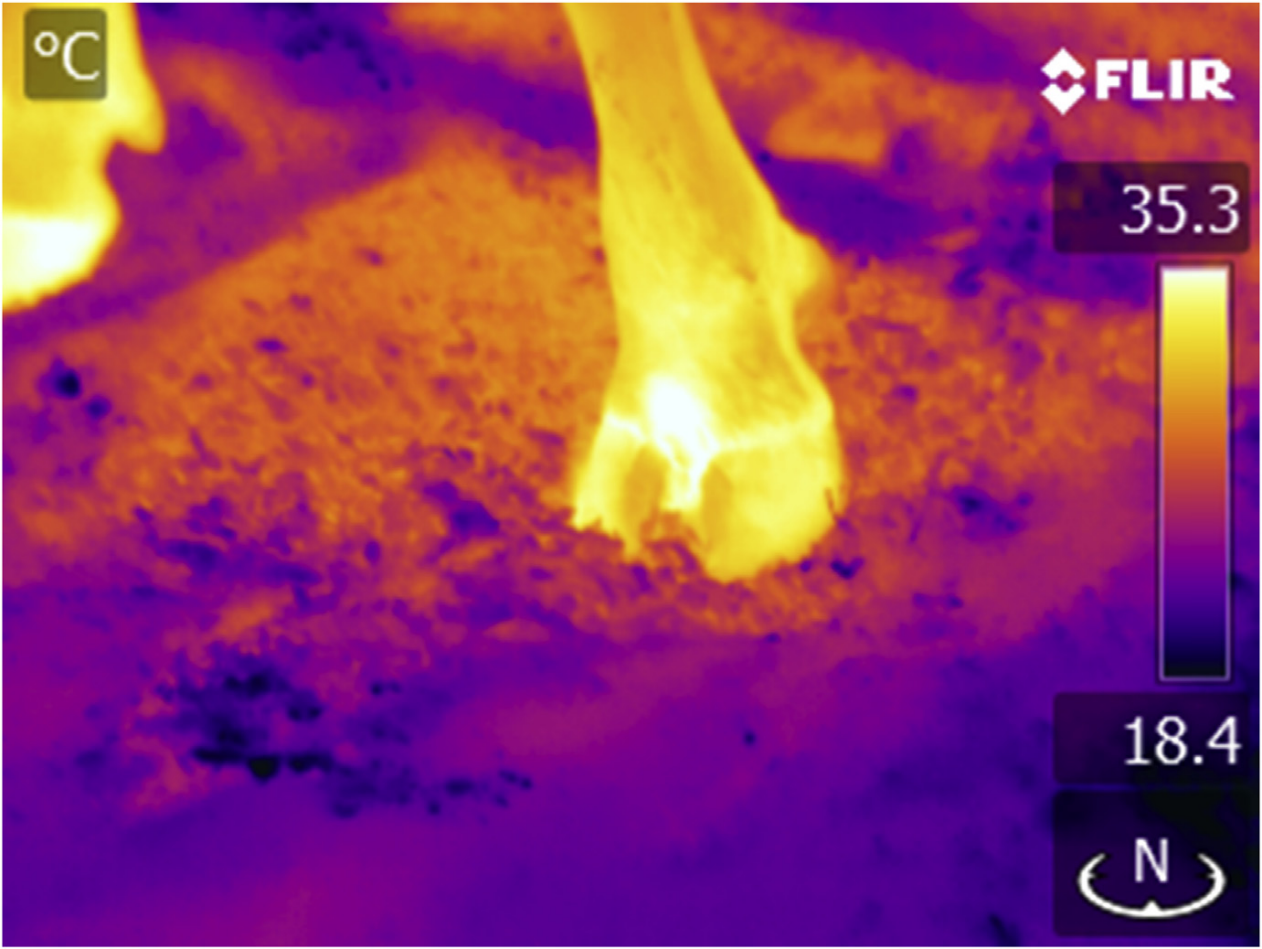
Image of a coronary band infrared scan site used with dairy cattle.

**Fig. 4:**
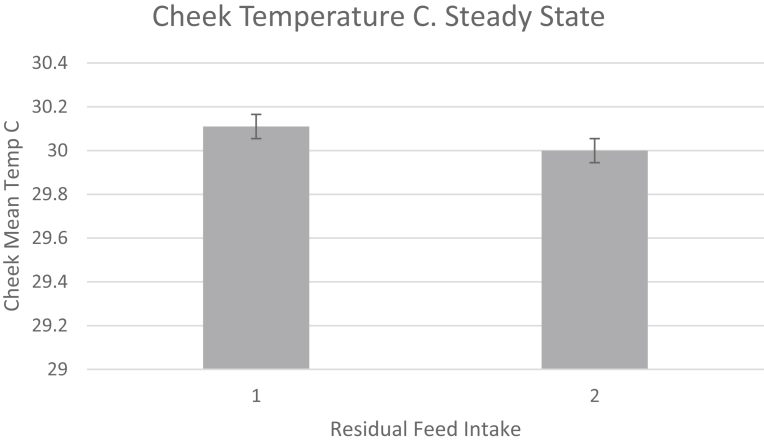
Cheek Temperature of Bulls in steady state (non induced) in high (1. RFI>0) and low (2. RFI<0) animals. Data is from 59 Bulls, non adjusted temperatures.

In terms of the time off feed measurements, the bulls and steers monitored at the University of Manitoba as well as the cows at the University of Alberta and the cattle at the Lacombe Research Centre were taken off feed for 24h immediately following their steady state feeding period. The bulls monitored at the Alberta test station site were taken off feed before the steady state RFI test. With respect to infrared images, lateral views of the animal's head were captured using a hand held IRT camera (FLIR Comp. Boston MA). An example of the lateral facial profile for the infrared images collected on all animals is shown in Figs. 1 and 2. For the cows monitored at the University of Alberta an image of the coronary band at the distal portion of the front legs was also captured as shown in Fig. 3. This approach has been documented by Montanholi et al. (2010). The areas of interest for measuring the mean “cheek” and “eye” images is identified. Images were collected form a distance of 2m while the animals were held in a conventional cattle chute. For the dairy cows at the University of Alberta the images were collected while the animals were in their tie stalls. Care was taken to standardize the operating procedures for focus, angle and image distance as well as atmospheric calibration for temperature and humidity. The hand held image collections for the University of Alberta cattle were conducted on all animals following an 18h period during which the cattle were held off feed. Infrared images were analysed using FLIR Researcher ® or ResearchIR ® software. A value for emissivity of 0.98 was used in the analysis. For the steady state thermal measurements collected at the University of Manitoba (Figs. 2 and 4) the lateral images were captured with an RFID driven automated infrared scan station as described by Schaefer et al. (2012). This system was positioned at a water bowl and used a FLIR A310 broad spectrum, high resolution (320 × 240 pixel) camera. FLIR Researcher® software was used to assess the thermal characteristics of infrared images.

### 2.2 Statistics

Data averages, standard deviations and parametric least squares analysis were conducted using Med Calc Version 9 software (2006). Response Operant Characteristic curves (ROC) were used to calculate the relationship between true positive or in-efficient animals (Sensitivity) and true negative or efficient animals (100-specificity) in the current study. This statistical approach is described by Zweig and Campbell (1993) and is commonly used in clinical biological data to determine optimal cut off values for selecting for or against specific traits (Schaefer et al., 2007). Response Operant Characteristic Curves were calculated in the present study using Med Calc Version 9 software (2006). Spearman Rank correlations were calculated according to methods described by Tuckman (1978) and Ramsey (1989).

## 3 Results

**Fig. 5:**
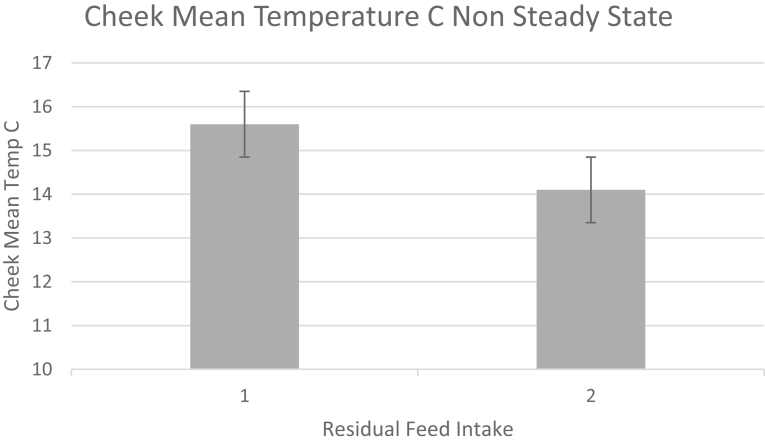
Cheek Temperature of Bulls in Non-Steady State (induced) in high (1. RFI>0) and low (2. RFI<0) animals. Data is from 59 Bulls, non adjusted temperatures.

The animals used in this study were predominantly yearling beef cattle weighing on average 545 kg. These beef animals gained on average 1.7 kg per day and consumed 10 kg dry matter per day on an *ad libitum* feeding regime. In addition, a significant number of beef cows and a smaller group of mature dairy cows were also included in the study. A total of 14 animals or 5.6% of the cattle displayed an orbital or eye maximum temperature of greater than 37 C (see Fig. 1 for example). These animals were represented by one animal in the University of Manitoba steer group, four animals from the Lacombe Research Centre group 1 and nine animals from the Lacombe Research Centre group 2 cattle. Data for these cattle were removed from the data set as these animals were deemed to be displaying temperatures indicative of illness likely depicting bovine respiratory disease (Schaefer et al., 2012). The range in environmental temperatures or delta T during the periods of testing for RFI are shown in Table 1. The delta T value for animals within a Thermal Neutral Zone was 20.15 C. This was in contrast to animals within a non thermal neutral zone displaying a delta T of 32.1C or nearly 40% greater. For all animals, the average cheek temperature for the non steady state test period was 28.9 ± 0.9 (SD) C. The off feed induction period was seen to reduce the cheek temperature in the animals as depicted in Figs. 4 and 5. With respect to the growth and growth efficiency measurements (Residual Feed Intake or RFI) the cattle demonstrated RFI values between -1.76 and 1.93 during the course of the study. For greater clarity, the animals consumed between -1.76 kg/d of feed less than predicted or up to 1.93 kg/d more than predicted (less efficient). The Spearman correlation for cattle examined within their TNZ demonstrated a significant correlation or rank relationship between RFI values and IRT non-steady state values collected with the hand held infrared camera r = 0.39 at P = 0.03. There was no statistically significant rank relationship for animals studied outside their TNZ (P > 0.05).

**Table 1:** Spearman rank correlation for RFI and IRT measurements for cattle within and not within a thermal neutral temeprature zone. Delta T represents the variation in daily average temperature during the RFI study.

Measurement	Animals Within Thermal Neutral Zone (TNZ)	Animals Not Within Thermal Neutral Zone (NTNZ)
Animals	85 Bulls, 16 heifers and cows	59 Bulls, 43 Steers and 33 cows
Delta T °C during the RFI 60–70 day test period	20.15	32.1
Mean Cheek Infrared T °C	28.4 ± 1.7	33.01 ± 0.94
FCE	5.64 ± 0.91(Bulls Only)	5.76 ± 0.72 (Bulls, Steers)
Spearman Rank RFI – IRT Cheek. r- value	0.39	0.09
Spearman Rank RFI -IRT Cheek. P - Value	0.03	0.60
N	101	135

**Fig. 6:**
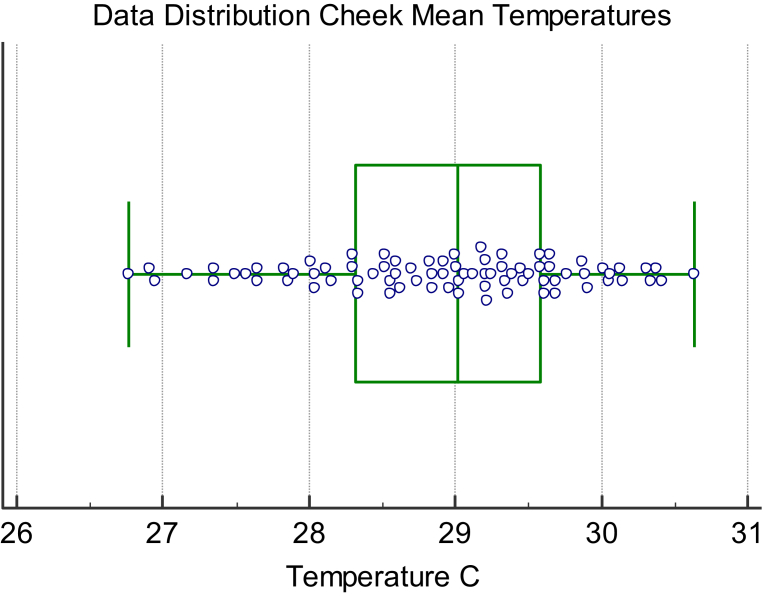
Box Distribution Graph for cheek infrared data for animals in their Thermal Neutral Zone.

**Fig. 7:**
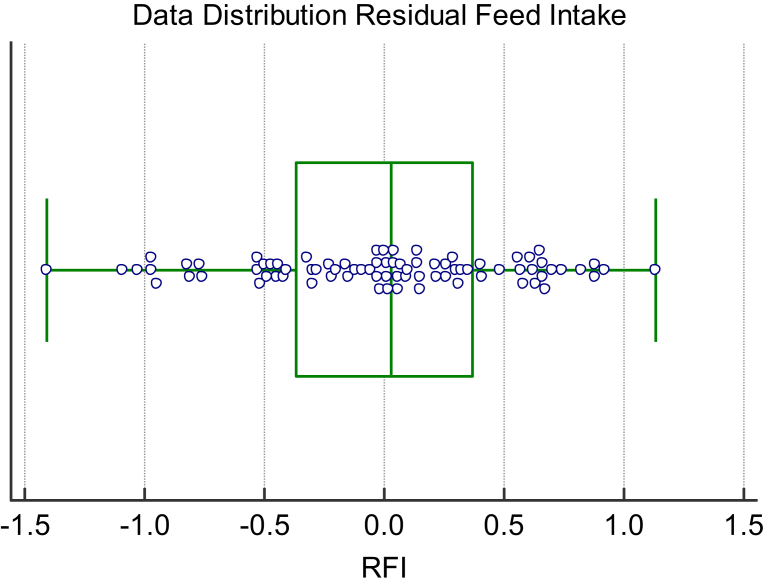
Box Distribution Graph for Residual Feed Intake (RFI) values for animals in their Thermal Neutral Zone.

The distribution of infrared thermal data and residual feed intake values for cattle studied at the University of Manitoba in a thermal neutral zone is shown in the distribution plots Figs. 6 and 7. Overall there were 44 animals displaying positive RFI values with an average value of 0.40 and 36 animals showing a negative RFI value with an average of -0.47. With respect to the IRT data, the animals with positive RFI values had a corresponding average post induction IRT value of 29.08 C. The animals with negative RFI values had a corresponding lower average post induction IRT value of 28.71 C and was significantly different than the animals displaying a positive RFI value (P < 0.05). This observation was consistent with the cows studied at the Lacombe Research Centre that were in a Thermal Neutral Zone showing an average adjusted temperature of 27.4 ± 1.8 C for animals with a negative RFI compared to the animals displaying a temperature of 28.5 ± 1.8 C for cattle with a positive RFI (P < 0.01). Also of interest was the observation from the preliminary dairy cow study at the University of Alberts again showing this same relationship. The two animals with a negative RFI value (average of -1.72) had an adjusted infrared value for the coronary band of 33.8 C compared to the two animals with a positive RFI average value of 1.3 and a coronary band temperature of 35.6 C. By contrast, for cattle not within a thermal neutral zone this relationship did not exist.

The use of a response operant characteristic assessment (ROC) for all the University of Manitoba cattle demonstrated an optimal cut off value (COV) of 29.3 C. This would produce a sensitivity (% selection for positive RFI) at 51.2% and a specificity (% selection for negative RFI) at 83.8%.

A pragmatic example of how the selection of animals based on the induction cheek temperatures could be used is shown in Table 2. Selection pressure for the upper and lower 10%, 20% and 30% would also select animals for differentiated RFI values (P < 0.05). The 50% selection level also shows a strong trend at P = 0.06.

**Table 2:** Examples of selection at different Cheek temperatures on RFI values. Values represent least squares means ±SD. Data is for U of M Bulls within a Thermal Neutral Zone.

	Lower Cheek Temp C	Higher Cheek Temp C	Probability	Lower Cheek Temp RFI	Upper Cheek Temp RFI	Probability
10%	27.2 ± 0.29	30.3 ± 0.18	<0.01	0.05	0.51	<0.01
20%	27.5 ± 0.42	30.1 ± 0.28	<0.01	0.11	0.41	<0.05
30%	27.8 ± 0.51	29.9 ± 0.33	<0.01	0.03	0.29	<0.05
50%	28.2 ± 0.63	29.6 ± 0.43	<0.01	-0.11	0.12	0.06

### 3.1 The impact of thermal neutrality

The data for the environmental conditions for the animals is shown in Table 1. As evident, the cattle in the non TNZ group were seen to experience environmental temperature changes or delta T values, more than 32 °C variation. Cattle in the TNZ were exposed to a much reduced variation in environmental temperature with a delta T value of 20 °C. Animals within a TNZ were also seen to show a significant ranking relationship between in a RFI and IRT values (P < 0.05) as would be expected and is similar to data seen in other studies (Schaefer et al., 2014). However, animals not in a TNZ did not show a significant ranking between RFI and IRT values.

Also of interest was the observation that the Feed Conversion Ratio or feed consumed/weight gain (kg) (FCR) were seen to be 2% higher in the cattle not in the TNZ (5.76 for animals not in the TNZ vs 5.64 for animals in the TNZ). This is logical and would be predicted in that the animals not in a TNZ would be expected to be using more feed energy for thermal regulation.

## 4 Discussion

Measuring the energy expenditure of an animal can be accomplished with many techniques (Colyn, 2013; Thompson, 2015). The gold standard in this regard is still likely the indirect calorimetry method. The use of feed intake measurements and growth has become popular in recent years in the cattle industry. However, as stated earlier this method is a steady state method and requires a considerable length of time. Perhaps more to the point, when utilized in outdoor environments the impact of changing environmental factors can render many of the measurement days unusable since they are outside the normal thermal neutral zone an animal would be adapted to. These factors plus the practical challenges of accessibility to equipment and cost often mitigate against the use in commercial herds of domestic animals.

Infrared thermography has been used to measure the energy expenditure in both human (Shuran, 1988, Kvedaras-Golos, 1980;) and animal models under steady state conditions (Scott et al., 2002; Colyn, 2013; Montanholi et al., 2008, 2010; Schaefer et al., 2005). This steady state approach has demonstrated good agreement between the measurement of animal efficiency, as represented by indirect calorimetry, residual feed intake (RFI) or feed conversion ratios and the infrared (IRT) data. This is, however, providing the animals were in a steady state condition within their estimated thermal neutral zone. In the present study an attempt was made to use a non-steady state or induction approach to measure and rank energy expenditure in a group of yearling bulls, yearling steers and cows. The logic again was that if challenged to conserve energy, via an acute time off feed period, animals better able to do so because of genetic capabilities would conserve energy by reducing their radiated energy loss to a greater extent than animals less able to do so. Indeed, as evidenced by the data presented (Figs. 4, 5, 6, and 7 and Tables 1 and 2) animals with lower measured and ranked RFI values also demonstrated a lower and ranked IRT value (P < 0.01).

Infrared thermography is a direct measurement of the energy exchange from an animal. The technology produces measurements which have been demonstrated to be significantly correlated with indirect calorimetry (Kvedaras-Golos, 1980; Shuran, 1988; Scott et al., 2002). The technique has also been demonstrated to show utility in ranking cattle for metabolic efficiency compared to RFI measurements under steady state conditions when the animals are within a thermal neural zone (Schaefer et al., 2005; Montanholi et al., 2010; Colyn, 2013; Thompson, 2015). The infrared thermography technique has also demonstrated the ability to rank animals for metabolic efficiency under non-steady state conditions (Schaefer et al., 2014).

Typical infrared scans collected on animals are shown in Figs. 1 and 2. The orbital image including the eye and the surrounding 1 cm of skin were used to determine animal health state as per the methods reported by Schaefer et al. (2007, 2012). In the current study all animals were well cared for and generally viewed as healthy by clinical scores conducted by pen checkers. In spite of this, fourteen animals or roughly 5.6% were removed from the data set because their eye or orbital max temperatures were above that seen for healthy animals. This suggests approximately 5.6% of the animals were suspect for health and/or stress reasons. These animals would have been expending energy in support of the immune system which would have biased the estimate of RFI. An additional note of interest with respect to the dairy cattle studied at the University of Alberta was that all of the cows were seen to be masticating or “chewing their cud” at the time of collecting the infrared scans. Hence, the exercise of the mandibular or jaw muscles was seen to be increasing the temperature of the facial scans. In this situation, the coronary band anatomical location was deemed as the appropriate scan site.

Of particular relevance also is the observation regarding the impact of the thermal neutrality during the study. As evident in Table 1, if cattle were outside the thermal neutral zone range during the previous 60–70 days of the RFI study then there was no relationship or ranking with the IRT or the direct measure of energy loss. The RFI values would have been biased due to energy expenditure for thermoregulation. However, if the animals were within their thermal neutral zone during the period of study then there was a statistically significant relationship between the direct measure of energy expenditure (IRT) and RFI. These are important factors to consider when measuring RFI.

The question thus arises as to how this procedure might be employed in a practical manner. In this respect there are two approaches suggested and demonstrated in the current manuscript. Firstly, the use of a Response Operant Characteristic curve or ROC method could be used to statistically identify cut off values (COV) for optimal designation into positive (inefficient) or negative (more efficient) animal groupings.

Secondly, and probably a more common approach from an animal herd management perspective would be to use a designated cut off value of practical significance to an animal management system. The example is provided (Table 2) using the highest and lowest 10, 20, 30 and 50% of the animal population for growth efficiency. The highest degree of selection pressure that a herd would experience would likely be the 20% (Quintal) level. Also, the highest and lowest 20% of the animals represent the least and most efficient animals for the herd respectively. The current method demonstrates that this type of ranking criteria would be effective in selecting animals displaying a 0.3 kg/d advantage in growth efficiency. An important factor to be kept in mind, however, is that the RFI assessment in itself is not perfectly accurate for reasons previously stated. Hence, comparing the IRT method to the RFI method would not be expected to produce perfect agreement.

Of additional significance, these IRT ranking methods could be employed within 24h. As stated previously, conventional methods for measuring and ranking animal energy efficiency such as indirect calorimetry and feed intake methods currently cost in the order of $300 per animal or more and often require in excess of 100 days to complete. Furthermore, the equipment is fixed in a specific location and not mobile. By comparison, a 24h ranking system using portable infrared equipment is estimated to cost in the order of a few dollars per animal. Furthermore, the infrared technology is much more available and able to be used in commercial herds. As a result, in addition to utility for herd replacement selections and estimated breeding value, the timely provision of this information would enable forecasting management decisions by monitoring animals at the start of a growth period. Animals could be identified, ranked and penned for efficiency such that fewer resources would be used in the production of food of animal origin. If a producer knew a specific animal was less efficient at the beginning of a feeding period for example they would be less likely to try to target that animal to a highly finished carcass quality grade, thereby saving energy feed input costs and time. These factors would have a significant impact on the use of carbon and energy in the animal industries.

## 5 Conclusions

The data suggests that the use of a non-steady state approach using infrared thermography for identifying metabolic efficiency in animals is a more rapid and cost effective method for identifying differences in energy utilization. The data also demonstrates the importance of thermal neutrality when monitoring metabolic efficiency irrespective of the methodology used. These findings on multiple sex, breed and age groups of cattle suggest the use of these infrared ranking methods merit further examination.

## Declarations

### Author contribution statement

A.L. Schaefer: Conceived and designed the experiments; Performed the experiments; Analyzed and interpreted the data; Contributed reagents, materials, analysis tools or data; Wrote the paper.

K. Ominski, S. Thompson: Conceived and designed the experiments; Performed the experiments; Analyzed and interpreted the data; Contributed reagents, materials, analysis tools or data.

G. Crow, C. Bench: Conceived and designed the experiments; Analyzed and interpreted the data; Contributed reagents, materials, analysis tools or data.

J. Colyn, A. Rodas Gonzalez, D. Maharjan, R. Bollum, J. Bassarab: Performed the experiments; Analyzed and interpreted the data; Contributed reagents, materials, analysis tools or data.

N. Cook, H. von Gaza: Analyzed and interpreted the data; Contributed reagents, materials, analysis tools or data.

### Funding statement

This work was supported by the Manitoba Agriculture Food and Rural Development, Alberta Livestock and Meat Agency, the Manitoba Beef Producers Saskatchewan Cattlemen's Association and Alberta Beef Producers. Financial support from the Manitoba Rural Adaptation Council Inc is also gratefully acknowledged.

### Competing interest statement

The authors declare no conflict of interest.

### Additional information

No additional information is available for this paper.
